# Appropriate health-seeking behavior and associated factors among people who had cough for at least two weeks in northwest Ethiopia: a population-based cross-sectional study

**DOI:** 10.1186/1471-2458-13-1222

**Published:** 2013-12-23

**Authors:** Meseret Senbeto, Sebsibe Tadesse, Takele Tadesse, Tesfahun Melesse

**Affiliations:** 1 Department of Internal Medicine, University of Gondar Hospital, Gondar, Ethiopia; 2 Institute of Public Health, the University of Gondar, Gondar, Ethiopia; 3 Department of Health Informatics, the University of Gondar, Gondar, Ethiopia

**Keywords:** Health-seeking behavior, Tuberculosis, Ethiopia

## Abstract

**Background:**

Tuberculosis remains the major debilitating public health problem in Ethiopia. However, studies to understand the patients’ perspectives on the illness and their health-seeking behavior have been few in the country. In this study, we seek to investigate the magnitude of appropriate health-seeking behavior and factors associated with tuberculosis among people who had cough for at least two weeks.

**Methods:**

A population-based cross-sectional study was conducted from July to October 2012 in Dabat, northwest Ethiopia. All people aged ≥15 years and had cough for at least two weeks were included in the study. Data collected by using a pre-tested and structured questionnaire were entered and cleaned using the Epi Info version 2002 statistical software. The statistical Package for the Social Sciences Version 16.0 was also employed for descriptive and logistics regression analysis.

**Results:**

Out of the 25,701 people aged ≥15 years surveyed, the proportion of people who had cough for at least two weeks was reported to be 843(3.3%). Appropriate health-seeking behavior towards tuberculosis was reported by 674(80.0%) of them. Factors significantly associated with health-seeking behavior for tuberculosis were being female [AOR: 0.56, 95%CI: (0.39-0.79)], high monthly real per capita income [AOR: 1.66, 95%CI: (1.15-2.38)], large family size [AOR: 0.50, 95%CI: (0.35-0.72)], and use of traditional-healing practices [AOR: 13.27, 95%CI: (9.10-25.41)].

**Conclusion:**

This study showed that the magnitude of appropriate health-seeking behavior during the event of chronic cough was high. However, this doesn’t mean that there will be no need for further strengthening of the intervention activities as significant proportions of the study communities still demonstrate inappropriate health-seeking behavior. So tuberculosis control programs need to emphasize factors, such as sex, family size, socioeconomic inequalities, and traditional-healing practices in resource-poor settings.

## Background

Tuberculosis (TB) has caused about 8.7 million new cases and 1.4 million deaths worldwide. It is second to the Human Immune Deficiency Virus (HIV) as a cause of death and ranks seventh among all illnesses as a cause of disability-adjusted life years lost [1].

Ethiopia is one of the high TB endemic countries in the world. The annual incidence and prevalence of all forms of TB has been 258 and 237 per 100,000 people, respectively [1]. A recent population-based survey conducted in Dabat district showed a prevalence of 174 sputum smear positive TB per a population of 100, 000 [2]. According to the 2011 report of the Federal Ministry of Health, the case detection rate at the national level was 36.8 [3].

Early diagnosis of active infectious cases and effective treatment are the cornerstones of TB control programs [4]. The World Health Organization (WHO) has recommended the Directly Observed Treatment Short- course (DOTS) approach as the most effective TB control strategy under the supervision of health care providers in resource-poor settings. The DOTS strategy has been adopted by many of the WHO member states, including Ethiopia. The Government of Ethiopia initiated a pilot TB control project based on the DOTS strategy in 1992. Since then the program has been subsequently scaled up in the country and implemented at the national level. The country’s DOTS geographic coverage has reached 100% according to the 2008 Federal Ministry of Health report [5].

Ethiopia follows the passive case finding strategy set by WHO for developing countries. In this situation patients’ understanding and response to the illness is mandatory to seek care and treatment [5]. Delayed diagnosis and treatment of the disease increases the risk of the spread of infection in the community, threatens the success of treatment, and increases the risk of multidrug resistance, relapse, and death. Knowledge about the health belief of patients can be used to tailor TB control programs in order to screen, diagnose, and treat patients more successfully. Studies on TB patients have shown that culture-based explanations, the use of traditional medicine, and religious- healing practices can be strong barriers to early diagnosis and effective treatment of TB [5]–[7]. However, only a few studies showed that patients’ health-seeking behavior is highly influenced by such socio-cultural factors in Ethiopia [5,7,8]. This study investigates the magnitude of appropriate health-seeking behavior and associated factors among people who had cough for at least two weeks in Dabat district, Ethiopia.

## Methods

### Study area, design and period

**Figure 1:**
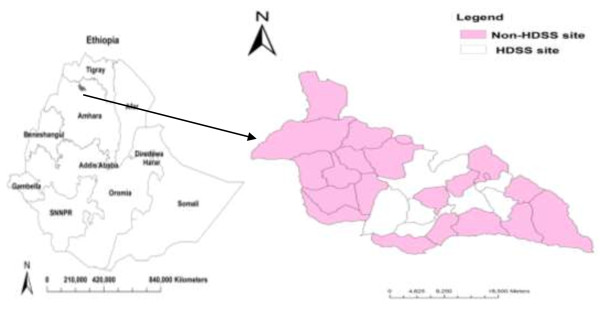
Map of Ethiopia and Dabat district: the shaded region shows the study area.

A population-based cross-sectional study was conducted in Dabat district, northwest Ethiopia from July to October, 2012. The district had an estimated population of 145,458 living in 27 rural and 3 urban kebeles (the smallest administrative unit in Ethiopia). Like the rest of the districts in the northern part of the country, the livelihood of the community largely depended on subsistence agriculture. Only two health centers delivered the directly observed chemotherapy (DOTS) service [9]. There was an on-going TB surveillance project at Dabat Health and Demographic Surveillance System (HDSS) hosted by the University of Gondar. The HDSS covered 10 randomly selected kebeles with a total population of about 46,165 [10]. This study was conducted on the non-HDSS kebeles where routine government health services, case detection, and treatment of TB under the National Tuberculosis and Leprosy Control Programs were available [2] (see map, Figure 1).

### Participants and data collection

All people aged ≥15 years and were living in the study area permanently (Government registered members) were considered as the source population and were included in the study if they were determined to have had cough for at least two weeks. Such people who were seriously sick and were on anti-TB treatment already were excluded from the study.

A pre-tested and structured interview questionnaire was used to collect data from the study participants to assess their health-seeking behavior for TB. The questionnaire contained detailed information on socio-demographic, behavioral, and environmental factors which were believed to affect the health-seeking behavior of the study participants (see Additional file 1). Data collectors performed house-to-house visits and asked the representatives of each household whether a person aged ≥15 years and had cough for at least two weeks at the time of the interview was present or not. Then data on health-seeking behavior for TB and associated factors were collected from each person who had with cough for at least two weeks. At the end of the interview, health education was given to the households with the intention of reducing stigma towards TB.

### Data quality assurance

The training of data collectors and supervisors emphasized issues such as data collection instruments, field methods, inclusion–exclusion criteria, and record keeping. The principal investigator and supervisors coordinated the interview process, spot-checked and reviewed the completed questionnaires on a daily basis to ensure the completeness and consistency of the data collected. They also conducted random quality checks by re-interviewing about 10% of the respondents. The interview questionnaire was pre-tested on 25 respondents who had characteristics nearly similar to people in Dabat district in order to identify potential problem areas, unanticipated interpretations, and cultural objections to any of the questions. Based on the pre-test results, the questionnaire was adjusted contextually. The entered data by the principal investigator and another independent individual were compared to check for any variations in results.

### Sample size calculation

Sample size was calculated on the Open-EPI sample size calculator software using a 5% level of significance, 4.2% prevalence of cough [2], the total adult population (aged ≥15 years) of 43,128, a 2% margin of error, a design effect of 2, and a non response rate of 10%. The final calculated sample size was 843.

### Sampling technique

We purposively considered 20 non-HDSS kebeles for sampling selection with the intention of excluding the effect of interventions by the TB surveillance project at the HDSS sites. Then 10 kebeles were randomly selected by the lottery method and the required sample sizes were allocated using probability proportional to size formula. Interviewees in the chosen kebeles were identified by house-to-house visits.

### Data management and statistical analysis

Data entry and cleaning carried out using the Epi Info Version 2002 statistical software, were analyzed on SPSS software package version 16.0. Descriptive statistics, such as frequency distribution, mean, and percentage were employed for most variables. Forward stepwise binary logistic regression analysis was done to assess the relative importance of the explanatory variables on the dependent variable (appropriate health seeking behavior). The odds ratio (OR) with a 95% confidence interval (CI) was used to test the statistical significance of variables.

### Operational definitions

#### Health-seeking behavior

Any action undertaken by individuals who perceive themselves to have health problems or to be ill for the purpose of finding an appropriate remedy [11].

#### Appropriate health-seeking behavior

The intention to seek diagnosis for TB at a medical facility or service in the event of a chronic cough [6]. In this study, those who mentioned to have visited health posts, health center and the hospital had appropriate health seeking behavior. Those who mentioned to have visited private clinics, pharmacies, Holy water, traditional healers and other sources had inappropriate health seeking behavior.

#### TB suspect

A person who had cough for at least two weeks [5].

#### Knowledge of TB

Those study participants who mentioned germs as causes of TB had good knowledge of TB [6].

#### Stigma towards TB

Eleven questions with 4 responses each (strongly disagree, disagree, agree and strongly agree) were asked to assess stigma. A total stigma score for TB was created by summing up the scores of all questions. Individuals who scored above the mean value were categorized as having high stigma towards TB [12].

#### Income

In this study, income referred to monthly real per capita income of the participants. Employed workers were asked their monthly salary, where-as farmers were asked the annual amount of cereal harvested and changed to Birr which was then divided by the months of the year. For the analysis, we used 500 Birr, which is the average urban and rural monthly real per capita total consumption expenditure set by the Federal Ministry of Finance and Economic Development of Ethiopia [13].

#### Family size

In this study we used 5 (the average fertility rate of Ethiopia) as an average household size [14].

#### Marital status

For analysis purposes, marital status was grouped into Single and Married. Single included never married, divorced, widowed, and separated.

### Ethical considerations

The study protocol was reviewed and approved by the Institutional Review Board of the University of Gondar via the Institute of Public Health. Government officials at various levels and community leaders were consulted and permission was obtained prior to data collection. Study participants were interviewed after informed written consent was obtained. Informed written consent regarding eligible participants below 18 years was obtained from parents or legal guardians. Individual records were coded and accessed only by the research staff (Additional file 1). Immediate referrals were arranged to the nearest health facility for those who had cough for at least two weeks.

## Results

### Socio-demographic characteristics of participants

Of the 25,701 people aged ≥ 15 years, the proportion of people who had cough for at least two weeks was reported to be 843(3.3%). Of them, 430(51.0%) were females. The mean duration of cough in the study participants was 19 days, with a range of 15 to 30 days. The mean age with a standard deviation of the participants was 44.4 ±1.6. The age of 605(71.8%) of the study participants was 35 and more years. The majority, 585(69.4%) of the participants were married. Five hundred seventy-two (68.7%) of the participants had no formal education. The majority, 768(91.1%) of the study participants were rural dwellers. More than two-thirds, 575(68.2%) had a monthly real per capita income of less than 500 Birr (Table 1).

**Table 1:** Socio-demographic characteristics of study participants in Dabat district, northwest Ethiopia, July to October 2012

**Variables**	**Number (N = 843)**	**Percent**
**Sex**		
*Male*	413	49.0
*Female*	430	51.0
**Age**		
*15-34*	238	28.2
*35-54*	348	41.3
≥*55*	257	30.5
**Marital Status**		
*Married*	585	69.4
*Single*	258	30.6
**Education**		
*Had no formal education*	585	69.4
*Primary*	226	26.8
*Secondary and above*	32	3.8
**Residence**		
*Rural*	768	91.1
*Urban*	75	8.9
**Income(in birr)**		
≤* 500*	575	68.2
*> 500*	268	31.8
**Family size**		
*< 5*	435	51.6
≥* 5*	408	48.4

### Health-seeking behavior of participants

Appropriate health-seeking behavior for TB was reported by 674(80.0%) of the participants. Out of these, 508(75.4%), 118(17.5%) and 48(7.1%) visited health centers, hospitals and health posts, respectively, when they had chronic coughs. Inappropriate health-seeking behavior for TB was reported by 169(20.0%) of the participants. Of these, 120(71.0%) used traditional-healing practices, 40(23.7%) did not know where to go, 7(4.1%) visited private clinics, and 2(1.2%) visited pharmacies in case of chronic coughs.

### Factors associated with health-seeking behavior

Table 2 presents the socio-demographic and behavioral factors which remained statistically significant in the bivariate and multivariate logistic regression analyses. According to this study, women were 0.56 times less likely to demonstrate appropriate health-seeking behavior for TB than males [AOR: 0.56, 95%CI: (0.39-0.79)]. The odds of having appropriate health-seeking behavior for TB among persons earning a monthly real per capita income of >500 Birr was 1.66 times higher than those earning a monthly real per capita income of ≤500 Birr [AOR: 1.66, 95%CI: (1.15-2.38)]. People from households of >5 people living together were 0.5 times less likely to demonstrate appropriate health-seeking behavior for TB than those from households of ≤5 people living together [AOR: 0.50, 95%CI: (0.35-0.72)]. People who would like to use traditional-healing practices in case of chronic cough were 13.27 times less likely to demonstrate appropriate health-seeking behavior for TB than those who would not [AOR: 13.27, 95%CI: (9.10-25.41)].

**Table 2:** Summary of logistic regression analyses on the relative effect of socio- demographic and behavioral factors on health-seeking behavior of study participants towards TB, in Dabat district, northwest Ethiopia, July to October 2012

**Variables**	**Health-Seeking**	**Crude OR**	**Adjusted OR**
	**Behavior**	**(95%CI)**	**(95%CI)**
**Sex**			
*Male*	413	1.00	1.00
*Female*	430	0.62 (3.6-7.00)	0.56 (0.39-0.79)
**Income(in birr)**			
≤* 500*	575	1.00	1.00
*> 500*	268	2.12 (1.49-2.99)	1.66 (1.15-2.38)
**Family size**			
*< 5*	435	1.00	1.00
≥ *5*	408	0.57 (0.39-0.79)	0.50 (0.35-0.72)
**Use of traditional-healing practices**			
*No*	723	1.00	1.00
*Yes*	120	18.74 (12.23-28.80)	13.27 (9.10-25.41)
**Smoking**			
*No*	805	1.00	1.00
*Yes*	38	2.18 (1.09-4.36)	2.01 (0.99-4.07)
**Cause of TB (Germs)**			
*No*	307	1.00	1.00
*Yes*	536	0.67 (0.47-0.96)	0.13 (0.02-1.08)
**Stigma towards TB**			
*Low*	210	1.00	1.00
*High*	633	0.66 (0.45-0.97)	0.17 (0.03-1.15)

## Discussion

In this study, the magnitude of appropriate health-seeking behavior during the event of chronic cough was 80.0% in the surveyed communities. This finding is higher than reports from India and Angola [6,15]. This may imply that the future burden of the disease in the communities is decreasing. The improvement might be due to continuous efforts to strengthen the health system infrastructure, increase public awareness, involve community health extension workers, build staff capacity, and step up the number of sectoral collaborations to prevent and control TB epidemic.

Like in some previous studies [16]–[18], women are less likely to have appropriate health-seeking behavior for TB than men in this study. This indicates that women are unaware of the importance of appropriate treatment service and its sources. One possible explanation for this could be that women have less access to information due to household work overload that includes caring for family members. Furthermore, in countries like Ethiopia, women are economically more dependent on their husbands, and their health is given less priority by family or community members. Also they are not autonomous in making household decisions including seeking healthcare in time of illness and mainly depend on their husbands’ decisions. Literature also indicates that women are more likely to have high social and perceived stigma associated with TB infection [19]. In order to alleviate this problem, TB control programs should focus on gender equity issues at all peripheral levels besides increasing women’s awareness to utilize the available resources, like maternal and child healthcare services. Furthermore, sociological research should be conducted to provide detailed evidence on the role of gender on TB healthcare-seeking behavior.

In this and other studies, high monthly real per capita income has had a significant association with appropriate health-seeking behavior for TB [20]–[22]. The reasons behind this may be that those who earn high monthly real per capita income may have more access to information by being furnished by the radio and television; the affordability of healthcare services may not bother them, either. In recommendation, TB control programs need to pay more attention to making TB care services accessible and affordable to the poor.

Less appropriate health-seeking behavior for TB was observed among participants from larger family sizes in this study. This might be due to the fact that those who had larger family members shouldered more family responsibilities and experienced severe socioeconomic hardships, which prevented them from visiting appropriate healthcare facilities for their illnesses.

In this study, participants who use traditional-healing practices in case of chronic cough are less likely to have appropriate health-seeking behavior towards TB than those who do not. In developing countries, it is estimated that about half the general public uses prolonged self-treatment involving traditional healing systems as the first step in the health-seeking process [23]–[25]. Such practices are common in areas where people try local healing practices before turning to DOTS services. This suggests that there is a need for intervention that encourages symptomatic individuals to seek the appropriate modern medical care as early as possible by establishing links to alternative healthcare providers.

The possible limitation of this study could be that we used self-reported data which might influence the reliability and validity of results as the majority of the study participants had no formal education. Although the HDSS sites were excluded from the study, they might have had some indirect impact on the health-seeking behavior of the study communities. We classified people who visit private healthcare sectors during the event of chronic cough as having inappropriate health-seeking behavior by considering the existing situations in the study area [26,27]. This may not hold true for other communities where the private healthcare sectors provide a range of medical services by involving highly trained medical specialists in adequately equipped diagnostic and treatment facilities.

## Conclusion

This study showed that the magnitude of appropriate health-seeking behavior during the event of chronic cough was high. However, this doesn’t mean that there will be no need for further strengthening of the intervention activities as significant proportions of the study communities still demonstrate inappropriate health-seeking behavior. So tuberculosis control programs need to emphasize factors such as sex, family size, socioeconomic inequalities, and traditional-healing practices in resource-poor settings.

## Competing interests

The authors declare that they have no competing interest.

## Authors’ contributions

MS: Initiated the research, wrote the research proposal, involved in data collection, data analyses and write up of the manuscript, ST: Involved in the data analyses and wrote the manuscript, TT: Involved in write up of the research proposal, the data analyses and write up of the manuscript, TM: Involved in write up of the research proposal, the data analyses and write up of the manuscript. All authors read and approved the final manuscript.

## Pre-publication history

The pre-publication history for this paper can be accessed here:

http://www.biomedcentral.com/1471-2458/13/1222/prepub

## Supplementary Material

Additional file 1 English version interview questionnaire and participant information sheet and consent form. Click here for file
