# Supplementary material for: Appropriate health-seeking behavior and associated factors among people who had cough for at least two weeks in northwest Ethiopia: a population-based cross-sectional study
Source: BMC Public Health. 2013 Dec 23;13:1222. doi: 10.1186/1471-2458-13-1222 (PMC3890638; doi:10.1186/1471-2458-13-1222)
Supplement: Additional file 1 — English version interview questionnaire and participant information sheet and consent form. [file 1471-2458-13-1222-S1.docx]

**English Version Participant Information sheet and consent form**

**A. Adult Consent form**

**Information to Study participants**

**Title of the study:** Health seeking behavior towards tuberculosis and its associated factors among adults with cough for more than two weeks.

**Introduction:** This information sheet and consent form is prepared for the aim of explaining the research project that you are asked to join by the group of research team.

Greeting-------------------------------------

My name is ________________________ I am a data collector for the research to be conducted by Meseret Senbeto, Sebsibe Tadesse, Takele Tadesse and Tesfahun Melesse, academic staff of University of Gondar, Institute of Public Health. They are conducting a study on Health-seeking behavior towards tuberculosis and its associated factors among adults with cough for more than two weeks at Dabat district in Northern Ethiopia. The information collected from this research project will be kept confidential and stored in a file, without your name. In addition, it will not be revealed to anyone except the investigators. All the responses given by participants will be kept confidential by using key and locked system like computer password whereby no one will have an access to it. You have full right to withdraw from this study at any time without a need to mention the reason why you wanted to withdraw. We value your input to make this study a successful one.

**Purpose of the study**

The main purpose of this study is to assess health-seeking behaviour towards tuberculosis and its associated factors among adults with cough for more than two weeks at Dabat district in Northern Ethiopia. The information that you will provide us will help us to improve TB prevention and control program in the study area in particular and in Ethiopia in general.

**Procedures**

If you agree to participate in this study, we will interview you about Health-seeking behaviour towards tuberculosis and its associated factors.

**Potential risks and discomfort of being in the study**

By participating in this research project, you may have some discomfort. There are no or minimal anticipated risk but taking time about 30 minute for interview.

**Benefits of being in the study**

There may not be direct benefits to you for giving us information for the study. If you have chronic cough you will be advised to visit the nearby health institution.

**Confidentiality and Privacy Protections**:

The records of this study will be stored securely and kept confidential. All publications will exclude any information that will make it possible to identify you as a subject.

**Contacts and Questions:**

If you have any questions about the study please ask me now. If you have questions later, or want additional information call the researchers conducting the study. If you have questions about the research please contact Meseret Senbeto**,** who is principal investigator of this study in the University of Gondar, at +251(0)918778495. You are making a decision about allowing participating in this study. Your signature below indicates that you have read or has been read to you the information provided above and has decided to participate in the study. If you later decide that you wish to withdraw to participate in the study, simply tell me. You may discontinue your participation at any time.

Name of study participant: _______________________

Signature: ____________Date:___________

Name of data collector______________Signature__________Date_________

**B. Parental information sheet and consent form for the Participation of Children**

**Aged 15-17**

**Title of the study:** Health seeking behaviour towards tuberculosis and its associated factors among adults aged ≥ 15 years with cough for more than two weeks at Dabat district, Northern Ethiopia.

**Introduction:** This information sheet and consent form is prepared for the aim of explaining the research project that you are asked to join by the group of research team.

Greeting-------------------------------------

My name is ________________________ I am a data collector for the research to be conducted by Meseret Senbeto, Sebsibe Tadesse, Takele Tadesse and Tesfahun Melesse, academic staff of University of Gondar, Institute of Public Health. They are conducting a study on Health-seeking behavior towards tuberculosis and its associated factors among adults aged ≥ 15 years with cough for more than two weeks at Dabat district in Northern Ethiopia. You are being asked to allow your child to participate in the study. This form provides you with information about the study. The researchers will also describe this study to you and answer all of your questions. Please listen the information below carefully and ask any questions you might have before deciding whether or not your child take part. The information collected from this research project will be kept confidential and stored in a file, without your name. In addition, it will not be revealed to anyone except the investigators. All the responses given by participants will be kept confidential by using key and locked system like computer password whereby no one will have an access to it. Your child participation is entirely voluntary; we respect both you and your child’s willingness to participate in this study. You or your child can refuse to participate without penalty or loss of benefits to which you are otherwise entitled. You can stop your participation at any time and your refusal will not impact current or future relationships with the institution that is going to carry out this study. To do so simply tell the researcher you wish to stop participation.

**Purpose of the study**

The purpose of this study is to assess Health-seeking behaviour towards tuberculosis and its associated factors among adults aged ≥ 15 years with cough for more than two weeks. The information that you will provide us will help us to improve TB prevention and control program in the study area in particular and in Ethiopia in general.

**Procedures**

If you agree your child to be in this study, we will interview about Health seeking behaviour towards tuberculosis and associated factors.

**Potential risks of being in the study**

There is no anticipated risk in this study unless the discomfort for 30 minute interviewing the chid may have.

**Benefits of being in the study**

There may not be direct benefits to your child for giving us information for the study. If your child have chronic cough, she/he will be advised to visit the nearby health institution.

**Confidentiality and Privacy Protections**:

The records of this study will be stored securely and kept confidential. All publications will exclude any information that will make it possible to identify him/her as a subject.

**Contacts and Questions:**

If you have any questions about the study please ask now. If you have questions later or want additional information call the researchers conducting the study. If you have questions about the research please contact Meseret Senbeto**,** who is principal investigator of this study in the University of Gondar, at +251(0)918778495. You are making a decision about allowing your child to participate in this study. Your signature below indicates that you have read or has been read to you the information provided above and has decided to allow your child to participate in the study. If you later decide that you wish to withdraw your child form the study, simply tell me. Your child may discontinue his/her participation at any time.

Name of child: __________________ Signature: _________Date:_________________

Name of Parent(s) or Legal Guardian: ___________Signature: _____Date:_________

Name of data collector ___________Signature: _____________Date_______________

Name of supervisor ____________Signature: ____________Date:_________

**English version participant questionnaire**

**Part I. General Information**

The information is taken from each adult who is ≥ 15 years and who have cough for two weeks and more than two weeks.

| **S.No** | **Question** | **Response** | **Code** |
| --- | --- | --- | --- |
| 101 | Field worker |  |  |
| 102 | Interview Date  (DD,MM,YYYY) |  |  |
| 103 | Kebele ID |  |  |
| 104 | House Hold No |  |  |
| 105 | House Hold head name |  |  |

**Part II: Socio-demographic Variable**

| **S.No** | **Question** | **Response** | **Code** |
| --- | --- | --- | --- |
| 201 | Age in complete years | **____________________** |  |
| 202 | Sex | Male……………………..…….1  Female……………………......2 |  |
| 203 | Marital status | Single ....................................1  Married ..................................2 |  |
| 204 | Education | Illiterate………………………….1  Primary level…………………...3  Secondary and above level…..4 |  |
| 205 | Monthly real per capita income income (in birr) | _______________________ |  |
| 206 | Residence | Urban ....................................1  Rural......................................2 |  |
| 207 | Family size | **________________** |  |
| 208 | Do you smoke | Yes …………………………...1  No ……………………….........2 |  |
| 209 | Where do you usually go if you are sick | Government Hospital…….....1  Health Center……………...…2  Health Post………………...…3  Private clinic……………….....4  Pharmacy…………………......5  Holy water………………..…...6  Traditional healer……….…....7  Other…………………………..8  Don’t know………………..….99 |  |

**Part III: Current illness and its duration**

| **S.No** | **Question** | **Response** | **Code** |
| --- | --- | --- | --- |
| 301 | Do you have cough for more than 2 weeks? | Yes………………….……..1  No ……………………...…2 | 303 |
| 302 | If yes to301, how long have it been since your current symptoms start? | In days |  |
| 303 | Do you have sputum? | Yes ………………………..1  No ………………………....2 | 307 |
| 304 | If yes to 303, for how long | In days |  |
| 305 | Do you have blood in sputum? | Yes ………………………...1  No ………………………….2 |  |
| 306 | If yes to 305, for how long? | In days |  |
| 307 | Do you have night sweats? | Yes…………………………1  No ……………………….....2 | 309 |
| 308 | If yes to307, for how long? | In days |  |
| 309 | Do you have loss of appetite? | Yes…………………………1  No…………………………..2 | 311 |
| 310 | If yes to 309, for how long? | In days |  |
| 311 | Do you have body weight loss? | Yes………………………....1  No……………………...…...2 | 313 |
| 312 | If yes to 311, for how long? | In days |  |
| 313 | Enlargement of lymph nodes? | Yes………………………….1  No……………………….......2 | 315 |
| 314 | If yes to313, for how long? | In days |  |
| 315 | Do you have fever? | Yes ……………………….....1  No ……………………….…..2 | 317 |
| 316 | If yes to 315, for how long? | In days |  |
| 317 | Do you have chest pain? | Yes ………………………....1  No …………………………..2 | 319 |
| 318 | If yes to 317, for how long? | In days |  |
| 319 | Do you have shortness of breathing? | Yes ………………………….1  No ……………………….......2 | 321 |
| 320 | If yes to 319, for how long? | In days |  |
| 321 | Have you ever been treated for TB? | Yes…………………………..1  No ..……………………….....2 |  |

**Part IV: Psychosocial related factors**

| **S.NO** | **Question** | **Response** | **Code** |
| --- | --- | --- | --- |
| 401 | Have you ever heard of TB? | Yes…………………………………..1  No……………………………………2 | **501** |
| 402 | If yes, where did you first hear about TB? | Health workers................................1  Former TB patients.........................2  Media..............................................3  Family/Relatives .............................4  Religious leaders.............................5  Teachers..........................................6  Other (specify )................................7 |  |
| 403 | Can TB be transmitted from one person to another? | Yes...................................................1  No.....................................................2  I don’t know.....................................99 |  |
| 404 | Which of the following do you think are causes of TB | Germs (bacilli)..................................1  Poverty.............................................2  Malnutrition......................................3  Witchcraft ........................................4  Sin/ curse.........................................5  Living with untreated TB patient.....6  Other (specify).................................7 |  |
| 405 | In your opinion how serious is TB? | Very serious……………………..….1  Somewhat serious……………...….2  Not very serious………………...….3  Don’t know………………………...99 |  |
| 406 | Please tell me some of the sign and symptoms of TB? | Cough..............................................1  Cough that last more than 2 weeks..............................................2  Coughing blood ..............................3  Severe Headache...........................4  Nausea...........................................5  Chest pain......................................6  Shortness of breath........................7  Weight loss.....................................8  Fever..............................................9  Weakness.....................................10  Other Specify ……………………..11  Don’t know …..…………………99 |  |
| 407 | How can a person get TB? | Handshaking ..................................1  Eating in same dishes.....................2  Through air, when TB patient coughs or sneezes...........................................3  Smoking..........................................4  Touching items in public places e.g. handles ,knobs...............................5  Other Specify………………………6  Don’t know....................................99 |  |
| 408 | How can a person prevent getting TB? (Multiple answers can be expected) | Avoid shaking hands........................1  Covering mouth and nose when coughing or sneezing………..…….2  Avoid sharing dishes .......................3  Washing hands after touching items in public places....................................4  Closing windows at home...............5  Through good nutrition....................6  Other specify………………………..7  Don’t know.....................................99 |  |
| 409 | In your opinion, who can be infected with TB? (Multiple answers expected) | Anybody...........................................1  Only poor people..............................2  Only homeless people......................3  Only alcoholics.................................4  Drug users.......................................5  Only people living with HIV/AIDS...6  Only people who have been in prison………….................................7  Other Specify……………….……….8  Don’t know......................................99 |  |
| 410 | If you had TB, others would think less of you | Strongly disagree………………...…1  Disagree …………….……….….…..2  Agree ………………………………...3  Strongly agree……………………....4  Don’t know…………………………99 |  |
| 411 | If you had TB, you would be ashamed/embarrassed | Strongly disagree………………...…1  Disagree …………….…………..…..2  Agree …………….……………….....3  Strongly agree……………………....4  Don’t know…………………………99 |  |
| 412 | If you had TB, others would avoid you | Strongly disagree………………….…1  Disagree……………...……………....2  Agree ………………………………....3  Strongly agree………………………..4  Don’t know………………………..…99 |  |
| 413 | If you had TB, you would have a problem of finding a partner for marriage even after cure | Strongly disagree……………………1  Disagree …………….………..……...2  Agree ……………..………………......3  Strongly agree………………………..4  Don’t know…………………………..99 |  |
| 414 | If you had TB, your partner would refuse to have sex with you | Strongly disagree…………………….1  Disagree ……………..…………….....2  Agree…………………………………..3  Strongly agree………………………..4  Don’t know………………………..…99 |  |
| 415 | If you had TB, you would be asked to stay away from a social group | Strongly disagree……………….……1  Disagree ……………..…………….....2  Agree ……………..………………......3  Strongly agree………………………..4  Don’t know…………………………..99 |  |
| 416 | If you had TB, you would not disclose even to a confident | Strongly disagree………..……..……1  Disagree ……………………………..2  Agree ………………………………....3  Strongly agree………………………..4  Don’t know…………………………..99 |  |
| 417 | If you had TB, you would think less of yourself | Strongly disagree……………………1  Disagree ……………..……………....2  Agree………………………………....3  Strongly agree…………………...…..4  Don’t know………………………….99 |  |
| 418 | If you had TB, you would make others affected by the disease | Strongly disagree……………………1  Disagree……………………………...2  Agree ………………………………...3  Strongly agree…………………...…..4  Don’t know………………………….99 |  |
| 419 | If you had TB, others would think less of your family | Strongly disagree……………………1  Disagree……………………………...2  Agree ………………………………...3  Strongly agree……………………....4  Don’t know…………………………99 |  |
| 420 | If you had TB, it would be a problem for your children | Strongly disagree………………...…1  Disagree ……………..……………...2  Agree ……………………………......3  Strongly agree……………………....4  Don’t know………………………....99 |  |

**Part V: Health Service Related Factors**

| **S. No** | **Question** | **Response** | **Code** |
| --- | --- | --- | --- |
| 501 | Do you know Public Health Facility or a Primary Health Care in your area? | Yes………………………...1  No…………………….........2  Don’t know………………99 |  |
| 502 | What do you think about public facility or a Primary Health Care in your area? | Excellent………………….1  Good…………….………...2  Poor……………………….3  Don’t know……………...99 |  |
| 503 | What do you think about the behavior of health care provider at public Facility or a Primary Health Care in your area? | Excellent………………….1  Good……………………....2  Poor……………………….3  Don’t know…………..….99 |  |
| 504 | Do you think Public Facility or a Primary Health Care in your area is well equipped? | Yes…………………..….…1  No………………….....…....2  Don’t know……………….99 |  |
| 505 | Do you think medicine is available at public facility or a Primary Health Care in your area? | Yes …………………….......1  No………………………..…2  Don’t know………………..99 |  |
| 506 | In your experience, what do you think about the waiting time at Public Health Facility or a Primary Health Care in your area? | 0-15 minutes…………..…..1  15-30 minutes……………..2  More than 30 minutes ……3  More than 1 hour …………4  Don’t know.........................99 |  |

**Part VI: Health Seeking behavior Questions**

| **S.No** | **Question** | **Response** | **Code** |
| --- | --- | --- | --- |
| 601 | How often do you generally seek health care at a clinic or hospital? (Check one.) | Twice a year or more……….…....1  Once a year……………………..…2  Less than a year but at least twice in the past………………………….……...3  Once in the past 5 years……..…..4  Not in the past year…………….…5  Other specify……………………...6 |  |
| 602 | Who would you talk to about your illness if you had TB? | Doctor or other medical worker….1  Pharmacy……………………….…2  Health extension worker………...3  Spouse…………………………….4  Parent……………………….……..5  Child(ren)……………………….…6  Other family member………..…....7  Close friend……………………….8  No one……………………………..9 |  |
| 603 | In case you had TB, where would you like to go to? | Governemnt Hospital……………..1  Health center…………………..…..2  Health post………………………....3  Private clinic……………………......4  Pharmacy…………………………...5  Holly water………………………….6  Traditional healer……………….….7  Other specify……………………..…8  Don’t know………………………...99 |  |
| 604 | Having gone to Government health facility, will you be satisfied with services? | Satisfied…………………………....1  Not satisfied…………………….....2  Don’t know……………………….99 |  |
| 605 | Having gone to Private health facility, will you be satisfied with services? | Satisfied…………………………...1  Not satisfied…………………….....2  Don’t know……………………….99 |  |
| 606 | Can you tell me the best source that could be used to make you aware about TB? | Television………………………...1  Radio…………………………..….2  Newspaper/Magazine……...…...3  Health provider……………....…..4  Relatives……………….…....…...5  Friends…………………….……..6  Booklets/Brochures………...…..7  Pharmacy………………………..8  Health extension workers…..…9  Don’t know………………….….99 |  |
